# Survey of quality control testing practices on low titer group O whole blood in the United States

**DOI:** 10.1111/trf.70074

**Published:** 2026-01-10

**Authors:** Mark H. Yazer, Skye Clayton, Christine M. Leeper, Philip C. Spinella

**Affiliations:** ^1^ Department of Pathology University of Pittsburgh Pittsburgh Pennsylvania USA; ^2^ Trauma and Transfusion Medicine Research Center, Department of Surgery University of Pittsburgh Pittsburgh Pennsylvania USA; ^3^ Department of Critical Care Medicine University of Pittsburgh Pittsburgh Pennsylvania USA

**Keywords:** leukoreduction, LTOWB, quality control, testing, US, whole blood

## Abstract

**Background:**

Use of low titer group O whole blood (LTOWB) continues to increase in the United States (US). This survey sampled the quality control (QC) practices among the largest blood collectors in the US.

**Methods:**

A survey on LTOWB collection, QC testing, and distribution was developed and electronically distributed to the chief medical officers of 16 large blood collectors in the US. Only complete survey responses were included.

**Results:**

The response rate was 10/16 (63%) representing approximately 80% of the US blood supply as estimated by the respondents. All of the respondents collected LTOWB from males and 7/10 (70%) also collected from never pregnant females. Eight out of nine (89%) centers that repeat donor anti‐A and ‐B titer testing do so on every donation, with 1/9 (11%) respondent testing previously low titer donors annually. The most common antibody titer threshold that defined low titer was <200 (6/10, 60%). The most common LTOWB collection method was with a platelet‐sparing filter in CPD 5/10 (50%). 7/10 (70%) respondents performed QC testing on LTOWB units; all of these respondents collected leukoreduced LTOWB and performed residual leukocyte count testing following leukoreduction. 6/7 (86%) performed residual red blood cell (RBC) recovery counts in the laboratory, 4/7 (57%) measured the unit's weight/volume, while 1/7 (14%) center measured the time taken for leukoreduction. The most often frequency of QC testing was monthly.

**Conclusion:**

Current practice at US blood suppliers for QC testing on LTOWB units was limited primarily to testing associated with leukoreduction and RBC counting.

**Abbreviations**
EDQM: European Directorate for the Quality of Medicine's and Health Care
ISBT: International Society of Blood Transfusion
LR: leukoreduced
LTH: life threatening hemorrhage
LTOWB: low titer group O whole blood
QC: quality control
RBC: red blood cell
REDCap: Research Electronic Data Capture
TQIP: trauma quality improvement
US: United States

## 1 INTRODUCTION

Low titer group O whole blood (LTOWB) is becoming increasingly popular for use in resuscitating patients with life threatening hemorrhage (LTH), including patients with non‐trauma etiologies of bleeding. According to a 2022 analysis of the trauma quality improvement (TQIP) database,^1^ there were 298 American trauma centers that were using LTOWB, and 70% of those centers were designated level 1 trauma hospitals. Among those hospitals were 33 trauma centers that were using LTOWB for children. LTOWB has many advantages over conventional component therapy^2^, ^3^ and, while awaiting the results of randomized trials comparing its efficacy to conventional component therapy, observational data has demonstrated potential survival benefit and safety among LTOWB recipients,^4^, ^5^ in particular those patients who received a relatively large volume of LTOWB as a fraction of the total volume of blood products transfused during the LTH.^6^, ^7^, ^8^, ^9^

However, the LTOWB that is supplied to hospitals across the country is not necessarily a homogenous product. No FDA regulations exist for quality control (QC) testing of whole blood. AABB Standards require establishing a center‐defined threshold for low titer of both anti‐A and ‐B.^10^ However, there are not any other specific Standards guiding the production of LTOWB in terms of whether it must be leukoreduced (LR), contain a minimum platelet concentration or pH, % hemolysis, or have minimum coagulation factor activities. As a consequence of not defining which QC tests to perform, the Standards do not specify the frequency of QC testing to be performed on LTOWB. As a result, AABB‐accredited blood collectors must decide for themselves what testing, if any, to perform and how often to conduct these tests.

Thus, to better understand the potentially different practices of producing LTOWB across the USA, and to inform the future practice of blood collectors that are planning to implement LTOWB collection, a survey of some of the largest blood collectors in the United States (US) was performed vis‐à‐vis their LTOWB collection, quality control testing, and distribution practices.

## 2 METHODS

A survey was developed to collect data on LTOWB collection, QC testing, and distribution in the US. Study data were collected and managed using Research Electronic Data Capture (REDCap) electronic data capture tools hosted at the University of Pittsburgh. REDCap is a secure, web‐based software platform designed to support data capture for research studies.^11^, ^12^ The survey responses were maintained in the REDCap database. The REDCap server was only accessible to two of the authors with electronic security certification. The survey was initially piloted by a blood center expert who was not involved with the survey's initial design and writing, and the survey was revised based on their recommendations.

A link to the final survey was sent by email to the chief medical directors of the 16 largest blood collectors in the USA. The invitation email contained an introductory statement about the nature of the survey as well as the link to the online survey. Because only one person at each blood center was approached to participate, redundant responses were not possible, although the task of responding to the survey might have been delegated by the chief medical director to someone else. Reminder group emails were sent to the medical directors once per week for 3 weeks starting about a week after the initial invitation was sent; personal emails were sent just before the survey closed to those who had not yet responded to encourage participation. The survey asked the respondents to report the number of LTOWB units distributed in 2024 because that was the most recent complete calendar year unless they started distributing LTOWB in 2025, in which case they were asked to extrapolate their current 2025 distributions for the full calendar year and to reply with their current procedures and policies for all other questions.

Incomplete surveys were not included in the final analysis. The survey response rate was calculated as the number of complete survey responses divided by the total number of medical directors who were contacted. Descriptive statistics were used to report the survey responses collected. Data were analyzed using Excel 365 (Microsoft, Irvine, CA).

The creation and dissemination of this survey did not qualify as human subjects research, thus the survey did not require review by the University of Pittsburgh's Institutional Review Board.

## 3 RESULTS

**FIGURE 1:**
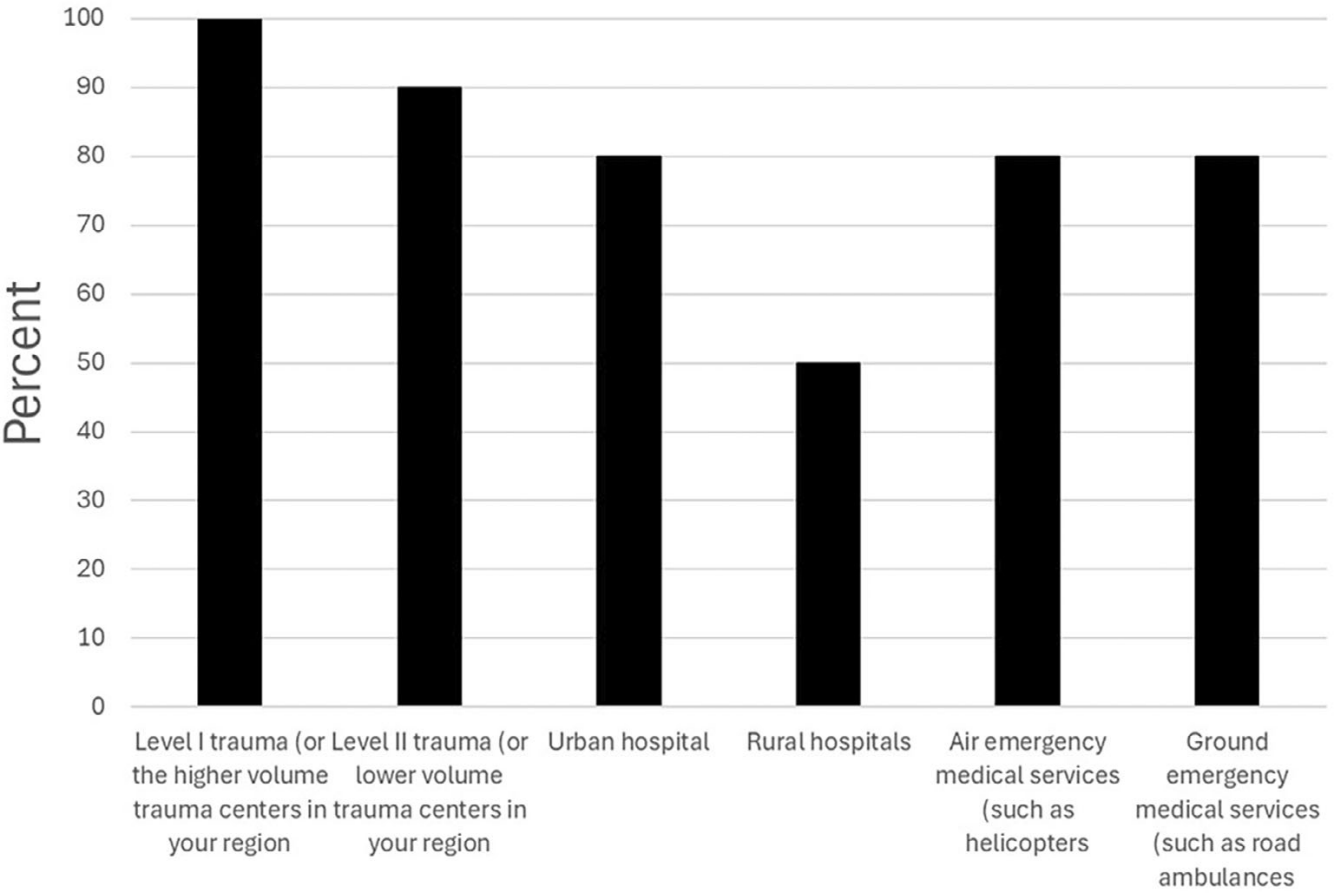
Nature of the facilities to which the respondents distribute LTOWB. The total of the percentages exceeds 100% because multiple replies were allowed.

Complete responses were received from 10/16 (63%) blood centers. At these centers, the median (range) number of RhD‐positive LTOWB units distributed in 2024 was 3680 (0–60,000), and 0 (0–20,000) for RhD‐negative LTOWB units. In fact, only 3/10 (30%) of the respondents distributed any RhD‐negative LTOWB units in 2024. Figure 1 demonstrates the nature of the facilities to which these centers distributed LTOWB.

**FIGURE 2:**
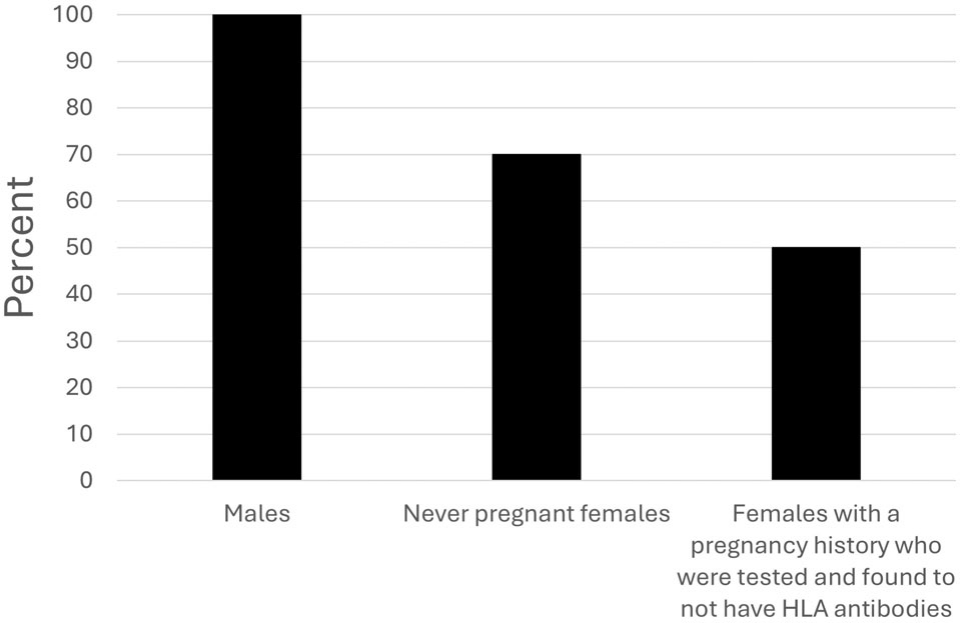
Nature of donors from which the respondents collect LTOWB. The total of the percentages exceeds 100% because multiple replies were allowed.

Figure 2 demonstrates that all of the respondents collected LTOWB from males and some collected LTOWB from other donors. At 8/10 (80%) of the respondents' blood centers, recent ingestion of aspirin and anti‐platelet medications would be exclusion criteria for donating LTOWB while recent ingestion of these medications would not be an exclusion at two blood centers.

Nine of the 10 (90%) respondents indicated that they test the donors' anti‐A and ‐B titers on subsequent donations following a successful LTOWB donation, while 1/10 (10%) center reported that once a donor tests as low titer, that status is assumed for subsequent donations. At 8/9 (89%) of the centers that repeatedly titer test whole blood donors, the donors are titer tested every time they donate whole blood, while at 1/9 (11%) center previously low titer LTOWB donors are titer tested annually.

The most common antibody titer threshold that defined a low titer donor was <200 (6/10, 60%), followed by <256 (2/10, 20%); one (1/10, 10%) center each used <150 and <100 as their titer thresholds. The titers were mostly performed using the manual tube method with immediate spin (7/10, 70%), while 1/10 (10%) of the centers used manual tube with room temperature incubation. At another center, manual anti‐human globulin was the test methodology, and at the remaining center, automated microplate testing using the Beckman Coulter PK4700 instrument was used to perform the titer.

In terms of LTOWB production, 3/10 (30%) of the respondents reported distributing only LTOWB that was leukoreduced using a platelet sparing filter in CPD. Three out of 10 (30%) responded that they only distributed non‐leukoreduced LTOWB in CPDA‐1, while 2/10 (20%) distributed both LTOWB that was leukoreduced with a platelet sparing filter in CPD and also non‐leukoreduced LTOWB in CPD. One (1/10, 10%) center reported distributing both LTOWB that was leukoreduced with a non‐platelet sparing filter in CPD and non‐leukoreduced LTOWB in CPD, and the remaining center (1/10, 10%) reported distributing LTOWB that was leukoreduced with a non‐platelet‐sparing filter in CPD. One center indicated that approximately 20% of their RhD‐positive LTOWB units were not leukoreduced while at that center 2% of their RhD‐negative LTOWB units were not leukoreduced.

In terms of QC testing of the LTOWB units, 7/10 (70%) respondents indicated that they performed such testing (Table 1). All seven of these centers distribute leukoreduced LTOWB and all perform a leukocyte count following leukoreduction. Most of the centers, 6/7 (86%) reported performing RBC recovery testing in the laboratory, and 4/7 (57%) also measured the LTOWB unit's weight/volume. One center (10%) measures the time to perform leukoreduction. Most centers perform QC testing on a monthly basis and the most common sample size was either an absolute number of 60 units or 5% of units produced. One site indicated that their QC testing is performed on an ongoing basis throughout the month until a predetermined number of LTOWB units has been tested; that number typically represents about 19% of the LTOWB units.

**TABLE 1:** Nature, frequency and number of LTOWB units undergoing quality control testing. Yes indicates that testing is performed. Blank spaces indicate testing is not performed.

Respondent	RBC recovery in the laboratory	Leukocyte count after leukoreduction	Unit weight/volume	Time required to perform filtration	Frequency of testing	How many units are used for QC testing?
1	Yes	Yes			Monthly	60
2	Yes	Yes			Monthly	60
3		Yes			Monthly	All LR LTOWB units^a^
4	Yes	Yes	Yes		ongoing throughout the month until a predetermined number of LTOWB units has been tested	~19% of LTOWB units
5	Yes	Yes	Yes		Monthly	5%
6	Yes	Yes	Yes	Yes	Monthly	60
7	Yes	Yes	Yes		Not specified	Not specified

^a^ This site indicated that they produced a small number of leukoreduced LTOWB units in 2024 and their QC testing was only performed on these units. This center's non‐leukoreduced LTOWB did not undergo QC testing.

## 4 DISCUSSION

This survey highlighted some of the LTOWB collection and QC practices from around the US. In this small sample, most suppliers collected and performed QC testing on LTOWB in the same manner but there were some interesting differences, particularly around donor exclusion criteria and antibody titer testing frequency. As expected, the extent of QC testing and the frequency of testing on LTOWB units was limited, likely due to the lack of specific testing requirements by the FDA and AABB. There are also no other regulations or standards for LTOWB QC metrics published internationally by organizations such as the International Society of Blood Transfusion (ISBT), European Commission, or European Directorate for the Quality of Medicine's and Health Care (EDQM).

The QC metrics for LTOWB could reflect quality and safety related assays for oxygen delivery and hemostasis. If implemented, the assays should also be standardized and simple for suppliers to perform. QC metrics for RBC production such as volume, hematocrit, hemoglobin, and percent hemolysis, in addition to the pre‐existing need to measure the white blood cell count after leukoreduction, could be incorporated into LTOWB QC metrics. QC metrics for the hemostatic capability of LTOWB are more complicated to determine since platelet count is not necessarily reflective of hemostatic function. Global measures of hemostasis such as viscoelastic assays could be considered, but there is no accepted threshold for minimum function that reflects quality. Ultimately, QC metrics should correlate with in vivo function and outcomes. Unfortunately, there is not high quality data that can inform this approach.

While this survey had a reasonable response rate, there were several responses that differed from the majority of the respondents, and with a larger group of respondents, perhaps additional variation would have been uncovered. Nevertheless, given which blood centers responded, it is estimated by the survey respondents' self‐assessed share of the marketplace that approximately 80% of the blood supply in the US was represented. The survey was intended to be primarily focused on LTOWB QC testing practices, so other questions about donor selection/qualification practices that might have varied between centers were not asked and could form the basis of a future survey.

Overall, the nature of QC testing performed on LTOWB units among these respondents generally revolved around leukoreduction, with some centers also testing RBC recovery and the weight/volume of the unit. These results were perhaps not surprising given that QC testing beyond that required following leukoreduction is not required for other blood products that are derived from a whole blood donation.

## CONFLICT OF INTEREST STATEMENT

PCS consults for Cerus, is on the scientific advisory board for Haima, Grifols, and Octapharma, and is a co‐founder and chief medical officer for Kalocyte. MHY is on the scientific advisory board for Hemanext, consults for Legacy Innovations, has given paid lectures for Terumo BCT, Grifols, and owns equity in Velico Medical, Inc.

## Data Availability

Research data are not shared.
